# Identification of Appropriate Reference Genes for qRT-PCR Analysis of Heat-Stressed Mammary Epithelial Cells in Riverine Buffaloes (*Bubalus bubalis*)

**DOI:** 10.5402/2013/735053

**Published:** 2013-01-28

**Authors:** Neha Kapila, Amit Kishore, Monika Sodhi, Ankita Sharma, Pawan Kumar, A. K. Mohanty, Tanushri Jerath, M. Mukesh

**Affiliations:** ^1^DNA Fingerprinting Unit, National Bureau of Animal Genetic Resources, Karnal, Haryana 132001, India; ^2^Biotechnology Division, Singhania University, Jhunjhnu, Rajasthan 333515, India; ^3^Animal Biotechnology Centre, National Dairy Research Institute, Karnal, Haryana 132001, India

## Abstract

Gene expression studies require appropriate normalization methods for proper evaluation of reference genes. To date, not many studies have been reported on the identification of suitable reference genes in buffaloes. The present study was undertaken to determine the panel of suitable reference genes in heat-stressed buffalo mammary epithelial cells (MECs). Briefly, MEC culture from buffalo mammary gland was exposed to 42 °C for one hour and subsequently allowed to recover at 37 °C for different time intervals (from 30 m to 48 h). Three different algorithms, geNorm, NormFinder, and BestKeeper softwares, were used to evaluate the stability of 16 potential reference genes from different functional classes. Our data identified *RPL4, EEF1A1,* and *RPS23* genes to be the most appropriate reference genes that could be utilized for normalization of qPCR data in heat-stressed buffalo MECs.

## 1. Introduction

The riverine buffaloes (*bubalus bubalis*) exhibit signs of great distress when exposed to direct solar radiations. This is generally attributed to their specific morphological, anatomical, and behavioural characteristics [1]. The effect of heat stress on mammary epithelial cell (MEC), the major cell type in lactating mammary gland, could be one of the prime factors responsible for lower milk production in animals. Understanding of expression profile of these cells from different livestock species during different physiological stages would provide molecular basis of heat stress specific transcriptomic response of mammary gland. Recently, few efforts [2, 3] have been made to unravel the transcriptional response of mammary gland to heat stress condition; still, the molecular mechanism of such responses are thought to be too complex to understand. qPCR is a common tool to determine the expression level of any target gene; for accurate quantification of expression level, there is a need to identify the appropriate reference genes under the particular experimental setup. Such approaches are helping a great deal to normalize the real time data for reliable interpretation of expression studies in different species [4–8]. In earlier studies, researchers have relied mostly on *GAPDH*, *ACTB, *and* RS18* as suitable reference genes [9–15]. However, Vandesompele et al. and Bustin et al. had shown that use of single reference gene can lower the reliability of expression data and strongly advocated use of multiple reference genes for each experimental setup [10, 12]. 

A number of studies have been conducted to identify the stably expressed candidate genes in different tissues of various livestock species such as pig, sheep, and bovines [5, 16–19]. These reports suggested that expression levels of commonly used reference genes vary considerably between different tissues and experimental conditions. Such variations reported in different studies [10, 17, 19–22] indicated that there cannot be set universal reference genes for all tissues or experimental conditions, and hence there is a need for identification of tissue specific stably expressed genes [23–31]. 

Environmental heat stress affects the mammary gland that results in low milk production or truncated milk production. Mammary epithelial cells (MECs) are responsible for converting most precursors into milk constituents and transporting them to the mammary lumen, the first line that gets affected by heat stress. As MEC ares the predominant cell types in lactating mammary gland, changes in their genes expression could provide an insight of the mammary gland mechanism. The present study was therefore undertaken to identify a panel of appropriate reference genes for normalization of transcriptional data of heat-stressed buffalo MECs. A total of 16 known reference genes, namely, glyceraldehyde 3-phosphate dehydrogenase (*GAPDH)*, beta-actin (*ACTB*), ubiquitously expressed transcript (*UXT*), ribosomal protein S15A (*RPS15A*), beta 2-microglobulin (*B2M*), alpha 2-microglobulin (*A2M*), ribosomal protein L-4 (*RPL4*), ribosomal proteinS18 (*RS18*), ribosomal protein L-22 (*RPL22*), ribosomal protein S9 (*RPS9*), ribosomal protein S23 (*RPS23*), hydroxymethylbilane synthase (*HMBS*), hypoxanthine-guanine phosphoribosyltransferase1 (*HPRT1*), GTP-binding protein (*GTP*), eukaryotic translation elongation factor 1 alpha 1 (*EEF1A1*), and ubiquitin C (*UBC*) belonging to different functional categories, were evaluated (Table 1). 

## 2. Materials and Methods

### 2.1. Sampling and Culturing of Mammary Epithelial Cells

Mammary tissue was obtained from an adult riverine buffalo and was immediately transported to laboratory in Dulbecco's Modified Eagle's Medium/Ham's F12 media (DMEM/F12, 1 : 1 mix) (Hyclone, Logan, UT, USA) containing antibiotics 100 U*⁄*mL penicillin-streptomycin (Hyclone). Five grams of tissue was washed with PBS (Ca^2+^-, Mg^2+^-free) (Hyclone) for several times until the solution was pellucid and without milk. The tissue sample was cut into 1 mm^3^ cubes and washed again. The smaller pieces of tissue were transferred to collagen-coated cell culture dishes (Corning, USA), containing DMEM/F12 supplemented with 10% fetal bovine serum (PAA), 100 U/mL penicillin-streptomycin (Hyclone), 5 *μ*L/mL insulin, 50 *μ*M hydrocortisone, 1 *μ*g/mL *β*-estradiol, 5 *μ*g/mL holotransferrin, and 1 *μ*g/mL Progesterone. (Sigma-Aldrich) and incubated at 37°C, 5% CO_2_. Initially, the basal media was replaced after every 12 h and then after every 48 h with fresh media until cells were visibly spread across the bottom of the culture dish. Cells were detached with 0.25% trypsin containing 0.02% EDTA (Sigma-Aldrich) and transferred to T75 culture flasks (Corning, USA). The process was repeated up to 10th passage, and pure mammary epithelial cells were obtained for further experimentation. 

### 2.2. Heat Stress Treatment of Mammary Epithelial Cells

The MECs were transported to collagen-treated 12-well plates in two sets with one plate assigned as control (kept at 37°C throughout the time course) and another plate as treated (exposed to 42°C). Initially, all the cells were incubated at 37°C with 5% CO_2_ for 30 m to stabilize the culture. Subsequently, the plate marked as treated was exposed to 42°C for 1 h to simulate the heat stress condition. After completion of 1 h, the cells were allowed to recover at 37°C with 5% CO_2_ and harvested at different time intervals (30 m, 2 h, 4 h, 8 h, 12 h, 16 h, 24 h, and 48 h). The samples from control plates were also harvested at same time points corresponding to the treated plates. After assessing the viability, cells were transferred to chilled Trizol reagent (Invitrogen Corp., CA) and stored at −80°C until RNA extraction. 

### 2.3. Isolation of Total RNA and cDNA Synthesis

Total RNA was extracted from MECs harvested at 30 m, 2 h, 4 h, 8 h, 12 h, 16 h, 24 h, and 48 h after heat stress using ice-cold Trizol (Invitrogen Corp., CA). RNeasy Mini Kit columns providing on with column digestion by RNAse-free DNase enzyme (Qiagen, Germany) were used to remove the traces of genomic DNA. Total RNA concentration and purity were measured using a NanoDrop ND-1000 spectrophotometer (NanoDrop Technologies). The quality check for all samples was performed using experion automated electrophoresis System (Biorad). All the extracted RNA samples were stored at −80°C and utilized within one month. cDNA was synthesized using 100 ng RNA, 1 *μ*L dT_12–18_ (Invitrogen Corp. CA), 1 *μ*L 10 mmol/L dNTP mix (Invitrogen Corp., CA), 1 *μ*L random primers (Invitrogen Corp., CA), and 10 *μ*L DNase-/RNase-free water. The mixture was incubated at 65°C for 5 min and kept on ice for 3 min. A total of 6 *μ*L of master mix composed of 4.5 *μ*L 5X First-Strand Buffer, 1 *μ*L 0.1 M DTT, 0.25 *μ*L (50 U) of SuperScript III RT (Invitrogen Corp., CA), and 0.25 *μ*L of RNase inhibitor (10 U, Promega, WI) was added. The reaction was performed in an Eppendorf Gradient cycler using the program: 25°C for 5 min, 50°C for 60 min and 70°C for 15 min. cDNA was then diluted 1 : 4 (v : v) with DNase-/RNase-free water. 

### 2.4. Primer Designing and Validation

To facilitate the real time PCR analysis, primers were either selected from the literature or designed using Primer Express 3.0 software (Applied Biosystem) with minimum amplicon size ranging between 50 and 115 bp and limited 3′ G+C content. Primer details for all genes are given in Table 2. To check the sequence specificity, primers were aligned against publicly available databases at NCBI and UCSC's Cow (*Bos taurus*) genome browser gateway using BLASTN. Prior to qPCR, primer specificity was further confirmed in a 20 *μ*L PCR reaction using the same protocol described for qPCR except for the final dissociation protocol. Five *μ*L of the PCR product was evaluated in 2% agarose gel stained with ethidium bromide. The accuracy of primer pairs was also ensured by the presence of a unique peak during the dissociation step at the end of qPCR. 

### 2.5. Real Time Quantitative PCR (RT-qPCR)

qPCR reaction was performed using LightCycler 480 instrument (Roche, Germany) in a 96-well white plate (Roche, Germany). Each reaction was comprised of 4 *μ*L diluted cDNA combined with 6 *μ*L of a mixture composed of 5 *μ*L 2X LightCycler 480 SYBR Green I master mix (Roche, Germany), 0.4 *μ*L each of 10 pmole forward and reverse primers, and 0.2 *μ*L DNase-/RNase-free water. For each gene, samples were analyzed in duplicate (technical replicates) along with 6-point relative standard curve and the nontemplate control. Following amplification conditions were used: 2 min at 50°C, 10 min at 95°C, 40 cycles of 15 s at 95°C (denaturation), and 1 min at 60°C (annealing + extension). A dissociation protocol with incremental temperatures of 95°C for 15 s plus 65°C for 15 s was used to investigate the specificity of qPCR reaction and presence of primer dimers. The qPCR expression data for each reference gene was extracted in the form of crossing points. The data was acquired using the “second derivative maximum” method as computed by the LightCycler Software 3.5 (Roche Diagnostics) and subjected for subsequent analysis. 

### 2.6. Evaluation of Expression Stability

The expression stability of each of the studied 16 genes was evaluated using three independent statistical applications: geneNorm [10], NormFinder [34], and Bestkeeper [35]. The geNorm, a Microsoft Excel-based application, was used to measure the expression stability as *M* value which is based on overall pairwise comparison among the reference genes. The calculated *M* value is inversely correlated to gene expression stability and ranks the reference genes accordingly. NormFinder, also a model-based approach, was also used to determine the optimal reference genes and the combination of two genes for a two-gene normalization factor with its corresponding stability value. Bestkeeper, another software used in the study, is based on pairwise comparisons of raw cycle threshold (Ct) values of each gene. The analysis assumes that the genes which are stably expressed should be highly correlated to each other. The 10 most stable genes as identified by geNorm analysis were considered for Bestkeeper analysis.

## 3. Results

**Figure 1:**
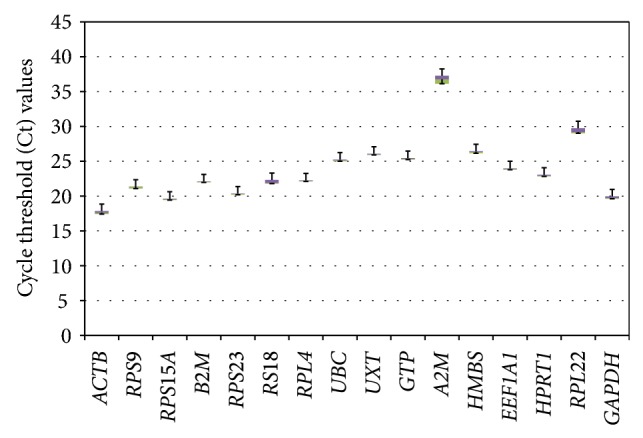
Overall expression pattern of 16 genes evaluated in heat-stressed MECs. The data is represented as qPCR cycle threshold (Ct) values of each gene in the box and whisker diagram. The median is shown as a dashed line across the box. The boxes represent median and 1st and 3rd quartiles ranges, while whiskers indicate maximum and minimum values.

The total RNA extracted from individual MECs samples exhibited high purity as determined by mean (±SEM) *A*_260/280_ ratio of 2.06 ± 0.014. The bioanalyzer-based RQ value of >8 also indicated sufficiently good quality of each extracted RNA. The qPCR performance for each gene in terms of coefficient of determination, (*R* ^2^), and efficiency of amplification (*E* = 10^−1/slope^) on the basis of slope of six-point standard curve are summarized in Table 2. The efficiency of PCR reactions ranged from 90.70% for *ACTB* to 131.37% for *B2M*. The characteristics of individual 16 genes based on their cycle threshold (Ct) values are shown as box whisker plot (Figure 1).

### 3.1. Analyses of Gene Expression Stability by GeNorm

**Figure 2:**
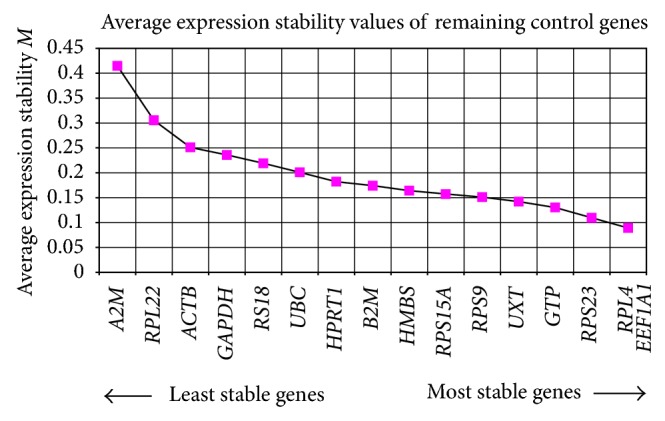
Average expression stability measures (*M* value) for reference genes.

The *M* value obtained under geNorm analysis ranged from 0.089 (*EEF1A1 and RPL4*) to 0.415 (*A2M*) (Table 3). All candidate genes performed well displaying *M* values below the accepted limit of 1.5. The genes were ranked from the most stable (lowest *M* value) to the least stable(highest *M* value): *RPL4*, *EEF1A1* > *RPS23* > *GTP* > *UXT* > *RPS*9 > *RPS15A* > *HMBS* > *B2M* > *HPRT1* > *UBC* > *RPS18* > *GAPDH* > *ACTB* > *RPL22* > *A2M* (Figure 2). Additionally, pairwise variation termed as “*V* value” was also calculated to determine the optimal number of genes necessary to calculate normalization factor. As suggested by Vandesompele et al., genes showing *V* value below the cutoff limit of 0.15 were selected as optimal number of genes for normalization [10]. In this analysis, we started with the two most stably expressed genes and then sequentially included less stably expressed genes. The lower the pairwise variation the better the combination of genes is. From this perspective, the *V* value was also calculated by adding the third and fourth less stable genes, that is, *V*3/*V*4 and *V*4/*V*5 combinations. The contribution of each gene to the variance of normalization factor ratio was calculated to illustrate the effect of adding or removing a particular gene from the final set of reference genes. 

**Figure 3:**
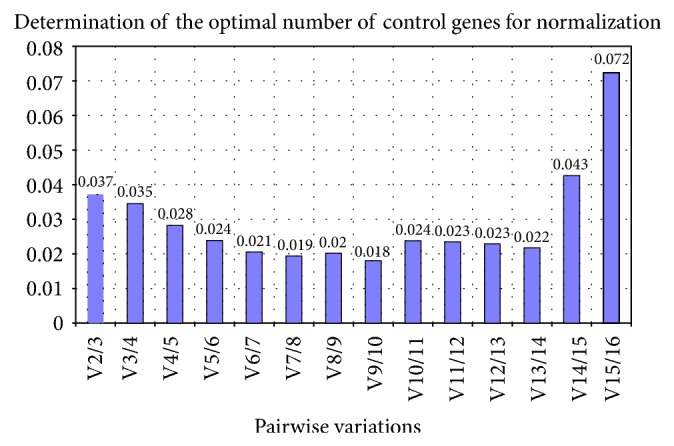
Determination of optimal number of reference genes for normalization by calculation of pairwise variation (*V*) of normalization factor ratios for different number of genes.

Our result showed that a combination of the two most stable genes (*RPL4* and *EEF1A1*) gave *V* value of 0.037 which is well within the acceptable limit (Figure 3). Similar approach has been used in a number of other studies to find out the suitable housekeeping genes [16, 19, 20, 36]. However, as the aim in such studies is to achieve an overall variation of less than 0.15 with minimum number of genes, *EEF1A1* and *RPL4* gene-combination could be appropriate to normalize the target genes expression in heat-stressed buffalo MECs. 

### 3.2. Analyses of Gene Expression Stability by NormFinder

**Figure 4:**
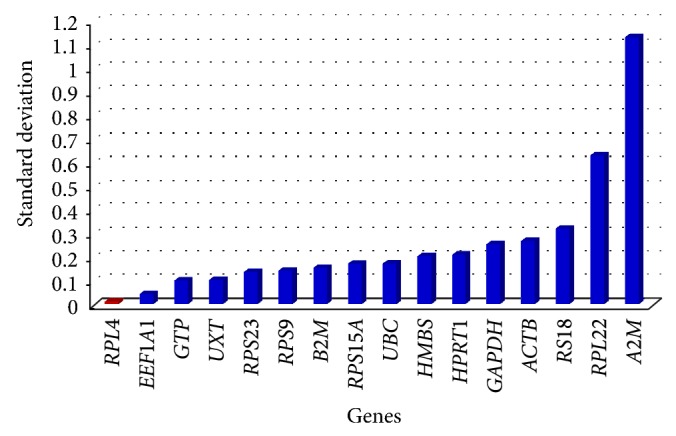
Bar plot showing gene variability in 16 candidate reference genes by NormFinder.

In addition to geNorm, we also utilized NormFinder software to find out expression stability of 16 genes. The analysis identified the same set of genes (*RPL4*, *EEF1A1*, *GTP*, *UXT*, *RPS23*) being most stable as revealed by geNorm only with slight change in their ranking order (*RPL4* > *EEF1A1* > *GTP* > *UXT* > *RPS23*) (Table 4, Figure 4). *A2M *and* RPL22* genes were found to be the least stable while *RPL4* as the most stable gene.

### 3.3. Analyses of Gene Expression Stability Using BestKeeper

BestKeeper analysis revealed the stability order as *RPL4*, *B2M* > *UXT* > *RPS15A, EEF1A1* >, and *RPS23 * with the crossing point standard deviation (SD [±CP]) value of 0.11, 0.11, 0.12, 0.13, 0.13, and 0.15, respectively (Table 5). These values were well within the acceptable range of fold change expression (<2). On the other hand, *RPS9* was the least stable with SD value of 2.06. In addition, intergene relation for the 10 most stable reference gene pairs was also estimated. The highly correlated reference genes were combined into BestKeeper index, and the correlation between each reference gene and BestKeeper was analyzed. The Pearson correlation coefficient (*r*), coefficient of determination (*r* ^2^), and the *P* values were estimated to describe the correlation between reference genes and BestKeeper index (Table 6). The best correlation was observed for *RPS9 *(*r* = 0.983) and *RPS23 *(*r* = 0.840) followed by *EEF1A1*, *RPL4*, *HMBS*, and *UXT* with values of 0.679, 0.567, and 0.565, respectively. 

The three different algorithms (geNorm, NormFinder and BestKeeper) employed in the present study to identify best suitable reference genes lead to cumulative result, and stability ranking showed almost similar trend. 

## 4. Discussion

For accurate interpretation of transcriptional studies, there is a widespread realization about the importance of suitable panel of reference genes for every species/tissue under study. In this study, our focus was to identify suitable reference genes in heat-stressed buffalo MECs as no such information is available in buffaloes. The findings of the present study will be a step forward to initiate transcriptional studies in this important dairy species of Indian subcontinent. The present analysis using different statistical applications clearly revealed a panel of the most stable reference genes (*RPL4, EEF1A1*, and *RPS23*) at different time points under heat-stressed condition of buffalo MECs. Our results for ranking of the most stable reference genes utilizing these different algorithms were quite comparable, albeit not identical. Similar to our findings, *RPL4* was also reported to be one of the most suitable genes in multiple tissues, namely, udder, muscle, liver, and kidney of water buffalo [37]. On the other hand, our results showed unstable expression with respect to the most commonly used reference genes, for example, *ACTB* and *GAPDH*. In the past, investigators have relied mostly on *GAPDH, ACTB*, and* RPS18* as reference genes [9–15]. Historically, *GAPDH *and* ACTB* genes have been used quite frequently as single control gene in more than 90% of the studies [21] and were considered good references for many years. But in the recent past, the expression of these reference genes has been shown to be affected by experimental condition [30, 38]. Several other studies have also shown variation in their transcription level [10, 17, 19–22] making them unsuitable for transcriptional studies. Proper evaluation of these two reference genes in any cell type or tissue of interest is therefore mandatory for correct interpretation of qPCR results.

In the present analysis, several genes were found to fulfill the criteria of suitable normalizer gene based on *M* values in geNorm (Figure 2), stability index in NormFinder (Figure 4), and Ct values in BestKeeper (Table 6). Overall, *RPL4, EEF1A1*,  and* RPS23* were selected to be the three most stable reference genes for normalization of gene expression data in heat-stressed buffalo MECs under *in-vitro* conditions. Vandesompele et al. [10] and Bower and Johnston [39] have also recommended the use of geometric average of the most stable reference genes for accurate normalization of qPCR data. As this is the first paper on testing the reference genes in heat-stressed buffalo MECs, our analyses were restricted to some of the well-known housekeeping genes reported in other livestock species. In conclusion, *RPL4, EEF1A1*,  and* RPS23* are the best reference genes for heat-stressed studies in buffalo MECs, and their geometric means would provide accurate normalization factor. For other experimental settings involving buffalo MECs, the use of these reference genes may be carefully evaluated as their expression may change in other specific experimental conditions. However, the panel reference genes identified in the present study would certainly be useful for accurate normalization of buffalo MECs expression data during heat challenge experiments.

## Figures and Tables

**Table 1:** General description, cellular localization, and biological functions for set of candidate genes evaluated as reference genes in the present study.

Gene symbol	Description	Cellular localization	Biological function/component
*A2M *	Alpha 2-microglobulin	Cytoplasm	A protease inhibitor and cytokine transporter

*ACTB *	*β*-actin	Cytoplasm	Cytoskeletal structural protein, nucleotide, and ATP binding

*B2M *	Beta 2-microglobulin	Golgi membrane, plasma membrane, early endosome membrane, extracellular region	Cytoskeletal protein, immune response, protein binding

*EEF1A1 *	Eukaryotic translation elongation factor 1 alpha 1	Cytoplasm	Translation elongation factor activity

*GAPDH *	Glyceraldehyde 3-phosphate dehydrogenase	Plasma membrane	Glycolytic enzyme, oxidoreductase in glycolysis and gluconeogenesis

*GTP *	GTP-binding protein	Cytoplasm and nucleus	Biogenesis of the 60S ribosomal subunit

*HMBS *	Hydroxymethylbilane synthase	Cytoplasm	Heme synthesis, porphyrin metabolism, transferase activity

*HPRT1 *	Hypoxanthine phosphoribosyltransferase	Cytoplasm	Purine synthesis in salvage pathway

*RPL22 *	Ribosomal protein L22	Cytoplasm	Component of the 60S subunit and encodes a ribosomal protein

*RPL4 *	Ribosomal protein L4	Cytoplasm	Component of the 60S subunit and encodes a ribosomal protein

*RPS15 *	Ribosomal protein S15	Cytoplasm	Protein synthesis/40S subribosome

*RPS18 *	Ribosomal protein S18	Cytoplasm	Component of the 40S ribosome

*RPS23 *	Ribosomal protein S23	Cytoplasm	Protein synthesis/40S subribosome

*RPS9 *	Ribosomal protein S9	Cytoplasm	Protein synthesis/40S subribosome

*UBC *	Ubiquitin C	Cytoplasm and nucleus	Protein degradation

*UXT *	Ubiquitously expressed transcript	Cytoplasm and nucleus	Transcriptional activation, ATP binding, microtubule binding, unfolded protein binding

**Table 2:** Gene name, GenBank accession numbers, primer sequences, annealing temperature (*T*_*a*_), amplicon length, and PCR efficiency for the studied genes.

Genes	Accession number	Primers 5′-3′ (forward, reverse)	*T*_*a*_	Amplicon size (bp)	PCR efficiency (%)^4^
*A2M* ^ 1^	CR452243	CACCCAGGACACAGTGGTAGC CCCTGAAGACTGGATGGTCAC	60°C	103	97.20

*ACTB* ^ 1^	AY141970	GCGTGGCTACAGCTTCACC TTGATGTCACGGACGATTTC	60°C	56	90.70

*B2M *	NM_173893	CTGCTATGTGTATGGGTTCC GGAGTGAACTCAGCGTG	60°C	101	131.37

*EEF1A1 *	BC105315	CATCCCAGGCTGACTGTGC TGTAAGCCAAAAGGGCATGC	60°C	101	115.50

*GAPDH* ^ 1^	BC102589	TGGAAAGGCCATCACCATCT CCCACTTGATGTTGGCAG	60°C	60	96.64

*GTP* ^ 1^	AK074976	CTTGGAATCCGAGGAGCCA CCTGGGATCACCAGAGCTGT	60°C	101	102.91

*HMBS* ^ 2^	BC112573.1	CTTTGGAGAGGAATGAAGTGG AATGGTGAAGCCAGGAGGAA	60°C	80	101.4

*HPRTI* ^ 3^	BC103248	GAGAAGTCCGAGTTGAGTTTGGAA GGCTCGTAGTGCAAATGAAGAGT	64°C	190	99.70

*RPL22* ^ 1^	BC114880	AAGATGGCGCCGAAGAAAG TTTCCCGAATCAAAAATTCCA	60°C	101	102.73

*RPL4 *	NM_001014894	TTGGAAACATGTGTCGTGGG GCAGATGGCGTATCGCTTCT	60°C	101	93.72

*RPS15A *	BC108231	GAATGGTGCGCATGAATGTC GACTTTGGAGCACGGCCTAA	60°C	101	119.08

*RS18 *	DQ222453.1	TTGCCTTTGCCATCACTG CTTGTATTGGCGTGGATT	60°C	158	129.97

*RPS23* ^ 1^	BC102049	CCCAATGATGGTTGCTTGAA CGGACTCCAGGAATGTCACC	60°C	101	115.5

*RPS9* ^ 1^	DT860044	CCTCGACCAAGAGCTGAAG CCTCCAGACCTCACGTTTGTTC	60°C	54	96.45

*UBC* ^ 2^	BE668033	TCCCTACCTGCATCATGTGC GGAATTTGGGCCAGTGCTC	59°C	71	104.71

*UXT* ^ 1^	CR452243	TGTGGCCCTTGGATATGGTT GGTTGTCGCTGAGCTCTGTG	60°C	101	99.74

^1^Bionaz and Loor [19],^2^Pérez et al. [32], and ^3^Hernandez et al. [33].

^4^qPCR efficiencies for each primer pair were calculated from six-point standard curves using fivefold dilution series of pooled cDNA from control and heat-stressed samples.

**Table 3:** Ranking of reference genes based on their expression stability.

Ranking order	Genes	*M* value
1	*EEF1A1 *	0.089
2	*RPL4 *	0.089
3	*RPS23 *	0.109
4	*GTP *	0.130
5	*UXT *	0.142
6	*RPS9 *	0.151
7	*RPS15A *	0.157
8	*HMBS *	0.164
9	*B2M *	0.174
10	*HPRT1 *	0.182
11	*UBC *	0.201
12	*RS18 *	0.219
13	*GAPDH *	0.236
14	*ACTB *	0.251
15	*RPL22 *	0.305
16	*A2M *	0.415

**Table 4:** Standard deviation (SD) and accumulated standard deviation (Acc. SD) for each gene analyzed through NormFinder.

Genes	SD	Acc. SD
*RPL4 *	0.0115	0.0115
*EEF1A1 *	0.0446	0.023
*GTP *	0.1029	0.0376
*UXT *	0.1056	0.0386
*RPS23 *	0.1397	0.0416
*RPS9 *	0.1457	0.0424
*B2M *	0.1578	0.0427
*RPS15A *	0.176	0.0434
*UBC *	0.1762	0.0432
*HMBS *	0.2081	0.0441
*HPRT1 *	0.2147	0.0446
*GAPDH *	0.2591	0.0463
*ACTB *	0.2735	0.0476
*RS18 *	0.3278	0.05
*RPL22 *	0.6443	0.0634
*A2M *	1.1554	0.0936

**Table 5:** Parameters based cycle point (CP) values for the 10 most stable housekeeping genes.

	*EEF1A1 *	*RPL4 *	*RPS23 *	*GTP *	*UXT *	*RPS9 *	*RPS15A *	*HMBS *	*B2M *	*HPRT1 *
*n*	9	9	9	9	9	9	9	9	9	9
GM [CP]	23.64	22.02	19.81	24.94	25.90	19.15	19.15	25.81	22.07	22.57
AM [CP]	23.64	22.02	19.81	24.94	25.90	19.54	19.15	25.81	22.07	22.57
min [CP]	23.31	21.79	19.42	24.54	25.70	10.27	18.80	25.31	21.86	22.14
max [CP]	23.91	22.30	20.02	25.27	26.30	20.87	19.43	26.06	22.37	23.00
SD [±CP]	0.13	0.11	0.15	0.16	0.12	2.06	0.13	0.18	0.11	0.20
CV [%CP]	0.57	0.52	0.76	0.64	0.47	10.54	0.67	0.71	0.49	0.89
min [x-fold]	−1.26	−1.18	−1.31	−1.32	−1.15	−470.28	−1.28	−1.42	−1.16	−1.34
max [x-fold]	1.21	1.21	1.16	1.25	1.31	3.30	1.21	1.19	1.23	1.35
SD [±x-fold]	1.10	1.08	1.11	1.12	1.09	4.17	1.09	1.13	1.08	1.15

*n*: number of samples; GM [CP]: geometric mean of cycling point; AM [CP]: arithmetic mean of CP; min [CP] and max [CP]: extreme values of CP; SD [±CP]: standard deviation of the CP; CV [%CP]: coefficient of variation expressed as a percentage on the CP values; min [x-fold] and max [x-fold]: extreme values of expression levels expressed as absolute x-fold over or under coefficient; SD [±x-fold]: standard deviation of the absolute regulation coefficients.

**Table 6:** Repeated pairwise correlation analysis among genes and with BestKeeper index (BI).

Genes	versus	*EEF1A1 *	*RPL4 *	*RPS23 *	*GTP *	*UXT *	*RPS9 *	*RPS15A *	*HMBS *	*B2M *	*HPRT1 *
*RPL4 *	*R *	0.893	—	—	—	—	—	—	—	—	—
	*P* value	0.001	—	—	—	—	—	—	—	—	—
*RPS23 *	*R *	0.841	0.800	—	—	—	—	—	—	—	—
	*P* value	0.005	0.010	—	—	—	—	—	—	—	—
*GTP *	*R *	0.790	0.592	0.718	—	—	—	—	—	—	—
	*P* value	0.011	0.094	0.029	—	—	—	—	—	—	—
*UXT *	*R *	0.776	0.426	0.519	0.793	—	—	—	—	—	—
	*P* value	0.014	0.251	0.151	0.011	—	—	—	—	—	—
*RPS9 *	*R *	0.712	0.608	0.766	0.351	0.464	—	—	—	—	—
	*P* value	0.032	0.082	0.016	0.355	0.207	—	—	—	—	—
*RPS15A *	*R *	0.507	0.250	0.592	0.788	0.634	0.363	—	—	—	—
	*P* value	0.163	0.518	0.094	0.012	0.067	0.337	—	—	—	—
*HMBS *	*R *	0.658	0.630	0.775	0.791	0.413	0.430	0.812	—	—	—
	*P* value	0.054	0.069	0.014	0.011	0.269	0.248	0.008	—	—	—
*B2M *	*R *	0.206	0.056	0.072	0.179	0.407	−0.042	0.384	0.168	—	—
	*P* value	0.593	0.885	0.855	0.646	0.277	0.915	0.308	0.666	—	—
*HPRT1 *	*R *	0.382	0.305	0.273	0.639	0.368	0.146	0.676	0.743	−0.013	—
	*P* value	0.312	0.423	0.478	0.064	0.329	0.708	0.045	0.022	0.977	*— *
BestKeeper Index	*R *	0.804	0.679	0.840	0.503	0.565	0.983	0.497	0.567	0.035	0.273
	*P* value	0.009	0.044	0.005	0.167	0.113	0.001	0.172	0.112	0.931	0.478
	*r* ^2^	0.646	0.461	0.706	0.253	0.319	0.966	0.247	0.321	0.001	0.075

*R*: Pearson correlation coefficient; *r* ^2^: coefficient of determination.
